# Impairments in Oxidative Glucose Metabolism in Epilepsy and Metabolic Treatments Thereof

**DOI:** 10.3389/fncel.2018.00274

**Published:** 2018-08-31

**Authors:** Tanya McDonald, Michelle Puchowicz, Karin Borges

**Affiliations:** ^1^School of Biomedical Sciences, Faculty of Medicine, The University of Queensland, Brisbane, QLD, Australia; ^2^Department of Nutrition, School of Medicine, Case Western Reserve University, Cleveland, OH, United States; ^3^Department of Pediatrics, University of Tennessee Health Science Center, Memphis, TN, United States

**Keywords:** glucose metabolism, pilocarpine, temporal lobe epilepsy, medium chain fatty acids, anaplerosis

## Abstract

There is mounting evidence that oxidative glucose metabolism is impaired in epilepsy and recent work has further characterized the metabolic mechanisms involved. In healthy people eating a traditional diet, including carbohydrates, fats and protein, the major energy substrate in brain is glucose. Cytosolic glucose metabolism generates small amounts of energy, but oxidative glucose metabolism in the mitochondria generates most ATP, in addition to biosynthetic precursors in cells. Energy is crucial for the brain to signal “normally,” while loss of energy can contribute to seizure generation by destabilizing membrane potentials and signaling in the chronic epileptic brain. Here we summarize the known biochemical mechanisms that contribute to the disturbance in oxidative glucose metabolism in epilepsy, including decreases in glucose transport, reduced activity of particular steps in the oxidative metabolism of glucose such as pyruvate dehydrogenase activity, and increased anaplerotic need. This knowledge justifies the use of alternative brain fuels as sources of energy, such as ketones, TCA cycle intermediates and precursors as well as even medium chain fatty acids and triheptanoin.

## Introduction

### Importance of Glucose Metabolism for Healthy Brain Function

Glucose metabolism is highly regulated due to the necessity to supply cells with energy critical for cell survival as well as specific cellular functions such as signaling. Any dysregulation of any pathway or inhibition of even a single enzyme in a given pathway can result in lactic acidosis and/or energy deficits. In humans with epilepsy and in several rodent epilepsy models, glucose metabolism is impaired. A deficiency in energy is likely to contribute to the generation of seizures, as the stabilization of membrane potentials and regulated neural signaling require high amounts of energy. This article first reviews the biochemistry of glucose oxidation and then provides an updated summary of the known mechanisms that contribute to the disturbance in oxidative glucose metabolism in the chronic epileptic brain (Tan et al., 2015; McDonald et al., 2017). These data provide the rationale to develop metabolic therapies as discussed in the last paragraphs.

**FIGURE 1:**
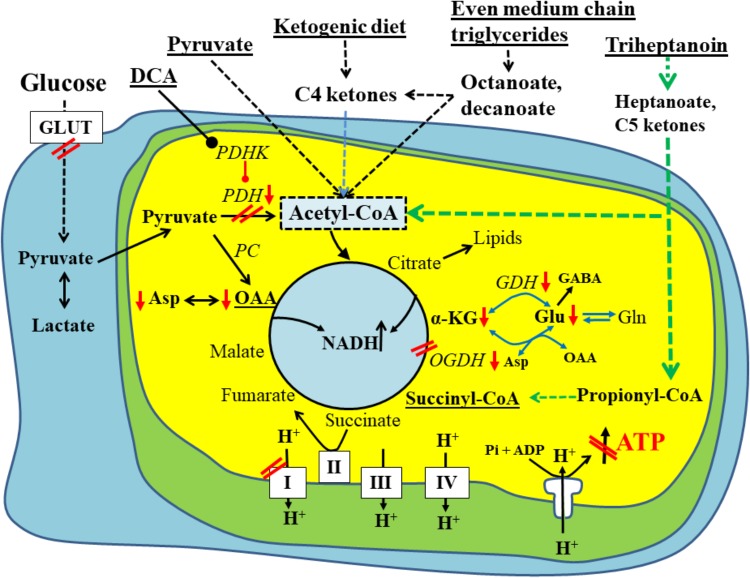
Schematic of biochemical impairments found in chronic epileptic tissue and potential treatments thereof. A brain cell under normal physiological conditions uses glucose (left) as the main fuel and produces pyruvate by glycolysis. Pyruvate can be metabolized to lactate in the cytoplasm, if there is an imbalance between glycolysis and TCA cycle activity. Otherwise, pyruvate is taken up by the mitochondria (inner yellow/green area) and is further metabolized by pyruvate dehydrogenase (PDH) to acetyl-CoA, which condenses with oxaloacetate to form citrate, the first intermediate of the TCA cycle. The TCA cycle produces NADH, FADH_2_, which can be used by the electron transport chain (complexes1-IV on the bottom of diagram) and ATP synthase (complex V) to ultimately produce ATP. Please note that the TCA cycle intermediates are also precursors for lipids and amino acids. The red arrows and double lines indicate reductions in metabolite levels, activities of pathways, enzymes or complexes or glucose uptake, which have been described in epilepsy in either human brains or animal models (see text for explanations). These impairments all limit ATP production by ATP synthase depicted in the diagram as a crossed out arrow in front of ATP in the lower right corner. Several metabolic treatment approaches for epilepsy are underlined and shown on the top and their metabolic effects are indicated. This includes PDH kinase inhibition (such as dichloroacetate) to increase PDH activity as well as direct fuelling with pyruvate, C4 ketone bodies and medium chain fats, which all directly produce acetyl-CoA and do not require PDH for entry into the TCA cycle. Please also note that pyruvate and heptanoate are both providing C4 TCA cycle intermediates, namely oxaloacetate via pyruvate carboxylase (PC) or succinyl-CoA, respectively. This anaplerosis may contribute to TCA cycling as well as biosynthesis of lipids and amino acids from TCA cycle intermediates. α–KG, α-ketoglutarate; GDH, glutamate dehydrogenase; GLUT, glucose transporter; OAA, oxaloacetate; OGDH, oxoglutarate dehydrogenase; PC, pyruvate carboxylase; PDH, pyruvate dehydrogenase; PDHK, PDH kinase.

The brain is a highly metabolically active organ and requires continuous supply of energy substrate delivery (Siesjo, 1978). Signal transduction uses around half of the energy required by the brain (see e.g., (Engl and Attwell, 2015)). In the brains of healthy people and most animals eating a standard diet, energy is largely produced from the metabolism of glucose. According to classic biochemistry, cytosolic glucose metabolism (glycolysis) generates small amounts of ATP, while oxidative glucose metabolism in the mitochondria generates the most ATP in cells, namely a net total of 2 ATP, 2 FADH_2_ and 8 NADH per glucose molecule in the TCA cycle and in the electron transport chain up to 2 and 3 ATP from FADH_2_ and NADH, respectively. Therefore, while eating a standard diet proper functioning of oxidative glucose metabolism and the electron transport chain is necessary for maintenance of normal brain activity. Several mechanisms described below can contribute to the impairments in oxidative glucose metabolism in the chronic epileptic brain, including presumed impairments in glucose transport and oxidative metabolism of glucose. Together, this limits the ability to produce ATP, which will promote destabilization of ion gradients and membrane potentials. Consequently, this can result in the generation of abnormal electrical activity and ultimately seizures (Samokhina et al., 2017). In addition, after seizures large amounts of energy are needed to re-establish ion gradients and membrane potentials as well as to repair damage. Further to this, entry of carbons into the TCA cycle is important to produce precursors for lipids, amino acids and proteins (**Figure 1**), essential building blocks for structural components of the brain as well as neurotransmission and learning. In the last paragraphs, we discuss several options of alternative fuels, which can overcome the new known mechanisms identified to limit oxidative glucose metabolism. These compounds directly enter the TCA cycle as acetyl-CoA and/or provide also C4 carbons substrates (anaplerosis) and are thus able to improve the production of energy and biosynthetic precursor production. Additional beneficial mechanisms, including anti-oxidant properties, and modulators of PDH activity are also discussed.

Other reviews and works regarding this topic are recommended and add more information about similar changes in neurodegenerative disorders and the contribution of hypometabolism to epileptogenesis (Zilberter and Zilberter, 2017), ((Malkov et al., 2018)and references therein) or focus more on oxidative stress (Kovac et al., 2017) or anaplerosis (Kovac et al., 2017). Please also note that we do not discuss the importance of glycolysis.

## Glucose Metabolism

### Glucose Transport and Glycolysis

Glucose is the main carbon source for energy generation and for biosynthesis of lipids and proteins, especially in brain. Thus its metabolism is both highly regulated and tissue specific. Cellular glucose entry is facilitated by the glucose transporter (GLUT) family, which consists of 14 GLUT proteins that are expressed in humans (Augustin, 2010; Thorens and Mueckler, 2010). The genes are part of the solute carrier family, called SCLT1-14. They have specific roles in cellular and/or whole-body homeostasis of glucose and other solutes, and are differentially expressed in specific tissue types. Of these, GLUTs 1-4 are the classical glucose transporters, which specifically transport glucose. Glucose transport in the brain is considered to be largely insulin-independent, with the high affinity GLUT1 expressed by the endothelial cells of the blood brain barrier and GLUT3 by neurons, while the lower affinity GLUT2 is expressed by astrocytes. In addition, in the last 20 years both mRNA and protein expression of insulin-dependent GLUT4 has been found, at least in the rodent brain (Unger et al., 1991; Brant et al., 1993; El Messari et al., 1998; Vannucci et al., 1998; Apelt et al., 1999). GLUT8 is another insulin-responsive isoform, which is exclusively expressed in neurons (Ibberson et al., 2000). Although the functional role of GLUT8 is still unknown, it is likely that it plays a role in increasing delivery of substrates to the brain when energy demands are increased.

Independent of cell type, after glucose entry, glucose is first phosphorylated by hexokinase (EC 2.7.1.1) producing glucose 6-phosphate. In the brain, glucose 6-phosphate typically enters the glycolytic pathway to generate NADH, ATP and pyruvate. The regulation of glycolytic flux is mainly regulated through the activity of phosphofructokinase (EC 2.7.1.11) (Hofmann, 1976), which hydrolyses ATP to phosphorylate fructose 6-phosphate to fructose 1,6-bisphosphate (Hofmann, 1976). Phosphofructokinase is allosterically activated by an increase in the ADP/ATP ratio (Uyeda, 1979; Zancan et al., 2008), while it is allosterically inhibited by increased levels of acetyl-CoA and the TCA cycle intermediates citrate (Uyeda, 1979). Also, the amounts of metabolites produced by the pentose phosphate pathway can regulate the activity of phosphofructokinase. Glucose 6-phophate can enter the pentose phosphate pathway by dehydration catalyzed by glucose 6-phosphate dehydrogenase (EC 1.1.1.49). While reducing NADP+, which is considered to be important for the production of reduction equivalents and antioxidant defense, the pentose phosphate pathway also provides C5 sugars for the synthesis of nucleic acids. The pathway also generates fructose 6-phosphate and glyceraldehyde 3-phosphate, which re-enter the glycolytic pathway (Kruger and von Schaewen, 2003; Wamelink et al., 2008). In specific cell types, such as hepatic cells and also astrocytes, glucose 6-phosphate can also be converted to glucose 1-phosphate by phosphoglucomutase and enter the glycogen synthesis pathway where glucose molecules are added to chains of glycogen for storage (Obel et al., 2012). Recent data showed that astrocytes produce glycogen largely during sleep, which is used as an energy source during the day (reviewed by O’Donnell et al., 2015). Moreover, recent findings suggest that neurons also exhibit glycogen stores and metabolism (Saez et al., 2014).

### TCA Cycle Metabolism and Oxidative Phosphorylation

Oxidation of glucose in brain is highly regulated and dependent on activities of enzymes and enzyme complexes. Any dysregulation or inhibition in their function, such as by phosphorylation or oxidative stress, can result in lactic acidosis and/or energy deficits. Pyruvate produced from glycolysis or other substrates, such as lactate, have three potential fates. In the cytosol, pyruvate can be transaminated to alanine via glutamate pyruvate transaminase, or it can be metabolized by lactate dehydrogenase to lactate (EC 1.1.1.27). The latter largely occurs under anaerobic conditions and oxidizes NADH to NAD+, which is needed to allow glycolysis to continue and produce energy. Finally, pyruvate can enter the mitochondria and produce even higher amounts of ATP than glycolysis through the TCA cycle and oxidative phosphorylation (Magistretti and Allaman, 2013). Pyruvate is transported into the mitochondrial matrix via mitochondrial pyruvate carriers, which were just identified in 2012 (Bricker et al., 2012; Herzig et al., 2012). Inside the mitochondria, pyruvate is converted to acetyl-CoA by the pyruvate dehydrogenase (PDH) complex to then enter the TCA cycle (Halestrap, 1975; Wieland, 1983; Vacanti et al., 2014). The complex consists of three subunits, namely PDH itself, the E1 enzyme (EC 1.2.4.1), the dihydrolipoyl transacetylase enzyme E2 (EC 2.3.1.12) and the E3 dihydrolipoyl dehydrogenase (EC. 1.8.1.4), the latter of which is also part of the oxoglutarate (α-ketoglutarate) dehydrogenase complex. Alternatively, pyruvate can be turned into oxaloacetate by pyruvate carboxylase (EC 6.4.1.1), providing the cycle with four carbon chain length intermediates (Patel, 1974). The activity of E1 pyruvate dehydrogenase is allosterically inhibited by an increase in the levels of ATP, NADH and acetyl-CoA (Pettit et al., 1975; Wieland, 1983). Furthermore, this complex is reversibly inactivated by the phosphorylation of the α-subunit of the complex, which catalyzes the rate-limiting reaction of the oxidative decarboxylation of pyruvate (Patel and Korotchkina, 2006; Seifert et al., 2006, 2007). The mammalian PDH E1 α-subunit component has three phosphorylation sites, all located on serine residues, and phosphorylation of any site by one of the pyruvate dehydrogenase kinase isoforms (PDK1-4) results in inactivation (Korotchkina and Patel, 2001). Pyruvate dehydrogenase phosphatases can dephosphorylate the serine residues and reactivate the enzyme (Patel and Korotchkina, 2006).

The acetyl-CoA produced from glucose or other substrates, such as lactate, ketone bodies or medium chain fatty acids (Kuge et al., 1995; Marin-Valencia et al., 2013; Zhang et al., 2015), condenses with oxaloacetate and enters the TCA cycle as citrate (Akram, 2014). The TCA cycle is a series of chemical reactions, which ultimately produce the high-energy metabolites NADH and FADH_2_ for the oxidative phosphorylation pathway (Fernie et al., 2004; Das, 2006). The activity of the cycle is highly regulated by substrate availability and product inhibition. However, three enzymes involved in the pathway, citrate synthase EC 2.3.3.1 (previously 4.1.3.7), isocitrate dehydrogenase (EC 1.1.1.41), and the oxoglutarate dehydrogenase complex (EC 1.2.4.2; EC 2.3.61 and EC 1.8.1.4) regulate the flux through the cycle based on energy needs (Fernie et al., 2004). Isocitrate dehydrogenase controls the carbon flow through the TCA cycle based on negative allosteric effects of NADH and ATP and the positive effectors isocitrate, ADP and AMP (Sazanov and Jackson, 1994). Oxoglutarate dehydrogenase is also regulated by the NAD/NADH ratio, and is a complex comprised of three isoforms (Lai and Cooper, 1986; McLain et al., 2011). The E3 component of oxoglutarate dehydrogenase, dihydrolipoamide dehydrogenase, is the same as the E3 component of pyruvate dehydrogenase, thus dihydrolipoamide dehydrogenase protein concentrations regulate both pyruvate entry into the cycle, along with TCA cycling rates (Patel and Roche, 1990). It is also important to note that many of the enzymes involved in oxidative metabolism are prone to inactivation by oxidative stress, including aconitase (EC 4.2.1.3) and the dehydrogenases.

Complete oxidation of a molecule of glucose occurs in the mitochondria by the electron transport chain, whereby the high-energy metabolites NADH and FADH_2_ donate electrons to electron acceptors and finally oxygen via a series of redox reactions. These reactions are carried out by four large multiprotein complexes located in the inner mitochondrial membrane of the mitochondria (Hroudová and Fišar, 2013). The electrons from NADH and FADH_2_ are moving step by step through complexes I, III, and IV of the electron transport chain. The energy released is used to transport protons from the mitochondrial matrix across the inner mitochondrial membrane to form a proton concentration gradient (Hroudová and Fišar, 2013). The movement of protons back into the mitochondrial matrix, driven by the proton-motive force, is coupled to the synthesis of ATP from ADP and P_i_ by the ATP synthase F_0_F_1_ complex, also sometimes named complex V (Hroudová and Fišar, 2013) (**Figure 1**). The functioning of the electron transport chain is highly sensitive to oxidative stress, as many of its components and cofactors are prone to oxidation.

### Anaplerosis – Refilling of the (Deficient) TCA Cycle Intermediate Pool

TCA cycle intermediates are precursors for the biosynthesis of lipids, amino acids and neurotransmitters. Therefore the pools of TCA cycle intermediates are constantly being turned over and if not replenished the pools will diminish. For example, citrate is the precursor for lipids, α-ketoglutarate produces glutamate and glutamine, while oxaloacetate can be turned into aspartate (**Figure 1**). For efficient production of energy, the TCA cycle is dependent on intermediates containing four or five carbons (via anaplerosis)(Owen et al., 2002). For example, acetyl-CoA can only enter the TCA cycle if oxaloacetate is present. There are several metabolites and reactions which can refill a (deficient) TCA cycle intermediate pool, which is termed anaplerosis. In astrocytes and oligodendrocytes, pyruvate can be carboxylated to produce oxaloacetate via pyruvate carboxylase. About 10-17% of brain pyruvate is metabolized by this pathway (Sonnewald, 2014). The four carbons gained can be transferred to neurons as glutamine. Neurons can then refill their TCA cycle from glutamine via metabolism to glutamate and then α-ketoglutarate catalyzed by glutaminase and glutamate dehydrogenase, respectively. In addition, the brain takes up glutamine from the blood (Bagga et al., 2014) and neurons can use glutamine/glutamate as fuel (Sonnewald, 2014; Divakaruni et al., 2017). Although the reaction catalyzed by glutamic oxaloacetic transaminase produces a TCA cycle intermediate, it is not truly anaplerotic, as oxaloacetate leaves the TCA cycle intermediate pool, while α-ketoglutarate is produced or vice versa. Another anaplerotic substrate in the brain is propionyl-CoA, which is produced by oxidation of isoleucine, valine, C5 ketone bodies (containing five carbons, see 4.1.2.) and potentially uneven medium chain fats (Brunengraber and Roe, 2006). Propionyl-CoA is carboxylated and ultimately forms the TCA cycle intermediate succinyl-CoA. The mRNA of the enzymes needed for this reaction, propionyl-CoA carboxylase and methyl-malonyl mutase have been found to be expressed in rodent neurons by *in situ* hybridization (Ballhausen et al., 2009; Tan et al., 2018). However, metabolic tracing of ^13^C-heptanoate indicates that propionyl-CoA predominantly produces glutamine, which indicates that astrocytic metabolism is responsible for the majority of this anaplerotic pathway (Marin-Valencia et al., 2013).

### Changes in Glucose Transport and Glycolysis Between and After Seizures

Based on ^18^F-flurodeoxyglucose positron emission tomography imaging (FDG-PET) or similar techniques using ^14^C-2 deoxy-glucose (2-DG), it has long been known that in human temporal lobe epilepsy (HTLE) as well as in chronic rodent epilepsy models interictal glucose metabolism in “epileptic foci/tissue” is impaired (Kuhl et al., 1980; Dube et al., 2001; Vielhaber et al., 2003). Reduced FDG or 2-DG transport and trapping in tissue has been termed hypometabolism and in many instances there is no correlations to neuronal loss (Kuhl et al., 1980; O’Brien et al., 1997; Dube et al., 2001; Vielhaber et al., 2003; Carne et al., 2004), Recent reviews show that FDG-PET-identified hypometabolism is useful to localize seizure foci also in other adult epilepsy forms and various pediatric epilepsy types [reviewed by (Burneo et al., 2015; Sarikaya, 2015; Zhu et al., 2017)]. Please note that these techniques can only provide information about glucose transport and the activity of hexokinase, as 2-DG and FDG are metabolized via hexokinase to produce 2-DG 6-phosphate or FDG 6-phosphate, respectively. Because these compounds are metabolically trapped, imaging on information about downstream metabolism cannot be obtained. The mechanism for the observed hypometabolism is still unclear, although we believe that reduced glucose uptake is likely, as in our mouse pilocarpine model hexokinase activity appeared to be unchanged in hippocampus (McDonald et al., 2017).

In addition, lactate concentrations are often elevated during and between epileptic seizures in human epileptic foci (During and Spencer, 1993; Cavus et al., 2005). Increases in lactate levels arise from an imbalance between the glycolytic and TCA cycle activity, either from the overproduction of pyruvate by increased glycolysis or from underutilization of pyruvate or both. The latter may be due to downstream blockades in metabolic steps, such as those involving pyruvate entry into TCA cycle as well as tissue hypoxia which both limit oxidative phosphorylation. Based on the presumed reduction of glucose transport in epileptic foci, increases in lactate may also indicate reduced pyruvate entry into the TCA cycle (see below).

In several animal models increases in brain lactate levels have been found after a period of seizure activity (During et al., 1994; Sacktor et al., 1966; Cornford et al., 2002; Urbach and Wellmer, 2013). After seizures elicited by flurothyl (a halogenated ether which blocks GABA_A_ receptor channels) in young and adult mice or the initiation of kainate-induced status epilepticus (SE) in rats there was an increase in the levels of hippocampal and brain lactate, respectively (Meric et al., 1994; Sacktor et al., 1966; McDonald and Borges, 2017). This increase was sustained throughout the 90 min kainate-provoked SE period (Meric et al., 1994). This elevation of lactate levels is thought to be caused by increases in the rate of glycolysis that are not matched with a rise in oxidative phosphorylation (Urbach and Wellmer, 2013; McDonald and Borges, 2017). Thus, the pool of pyruvate increases within the cell driving lactate dehydrogenase to metabolize it to lactate. After a five min flurothyl-induced seizure our laboratory found no other changes in the maximal activities of hippocampal glycolytic enzymes measured, except for a 30% decrease in phosphofructokinase activity, which we will not discuss here as this review focusses on oxidative metabolism of glucose. In addition, we found that total citrate, glutamate and aspartate levels were not significantly altered (McDonald and Borges, 2017). This again supports the notion that during seizures the increase of the glycolytic flux is not linked with an upregulation in the TCA cycle activity (Edmond et al., 1987). Interestingly, there are indications that lactic acidosis may be involved in the termination of seizures via multiple mechanisms. This is reviewed by (Yang et al., 2013) and will not be discussed here.

In rodent models of chronic epilepsy, in which animals show spontaneous seizures, however, brain lactate levels are highly variable and several studies dispute the reports of increased lactate concentrations (Melo et al., 2005; Dubé et al., 2010; Smeland et al., 2012, 2013). Taken together, these studies show that glucose metabolism is perturbed during seizures and in rodents of chronic epilepsy models. This may be caused by recurrent seizures, but also is likely to contribute to seizure development.

### Impairments in the TCA Cycle and Electron Transport Chain and Need for Anaplerosis

Understanding the metabolic mechanisms of impairments of glucose metabolism in human epilepsy and animal models has improved our knowledge to design therapies targeting these metabolic defects. In patients, impaired oxidative glucose metabolism has been shown in surgically removed epileptic foci (Vielhaber et al., 2003; Kann et al., 2005). For example, Vielhaber et al. (2003) showed that the reduction in interictal FDG-PET correlated with decreased glucose oxidation in the CA3 area measured by respirometry in hippocampal slices obtained from surgery specimen. After electrical stimulation tissue from both TLE patients and rats in the chronic stage of the pilocarpine model showed smaller increases in NAD(P)H fluorescence after an initial drop that indicate impaired NAD(P)H reduction in epileptic tissue (Kann et al., 2005). As the negative mitochondrial membrane potentials and activation-dependent mitochondrial depolarizations were normal in neurons, it is likely that mitochondrial enzyme activities are impaired, which was recently confirmed by our group in mice. Alternatively, dysfunctions on the level of astrocytic glycolysis and/or neuronal-astrocytic metabolic coupling is possible.

Animal studies have allowed further insight into the changes of glucose oxidation by the TCA cycle, revealing decreases in PDH activity and increased need for anaplerosis [see below and (Kovac et al., 2013)]. The increase in neurotransmission, and the increase in neurotransmitter release during a seizure (Kovács et al., 2003) is associated with an increased metabolic need due to neurotransmitter reuptake and clearance along with depletion of several pools of TCA cycle intermediates. Oxaloacetate and α-ketoglutarate, two intermediates of the TCA cycle, are precursors for the synthesis of aspartate and glutamate, respectively. The quantification of these two amino acids and the respective neurotransmitters in several chronic animal models of epilepsy have shown that the concentrations are reduced in the “epileptic” brain (Melo et al., 2005; Alvestad et al., 2008; Willis et al., 2010; Smeland et al., 2013). This suggests that the total levels of the TCA cycle intermediates are also decreased, which was corroborated in the forebrain of mice during the chronic stage of the pilocarpine-SE model (Willis et al., 2010), but not in hippocampus (McDonald et al., 2017). There has been some concern that the observed reductions in metabolite levels from both human and animal tissues are largely due to cell loss. However, several studies have found no correlation between concentrations of the neuronal metabolite N-acetyl aspartate and cell death, indicating that metabolic changes found in chronic epilepsy models are likely due to impaired neuronal metabolism (Kuhl et al., 1980; Dube et al., 2001; Vielhaber et al., 2003). In summary, most of these studies provide evidence for decreased activity of the TCA cycle and increased anaplerotic needs due to decreases in the levels of TCA cycle intermediates (**Figure 1**).

#### Pyruvate Dehydrogenase Activity

**FIGURE 2:**
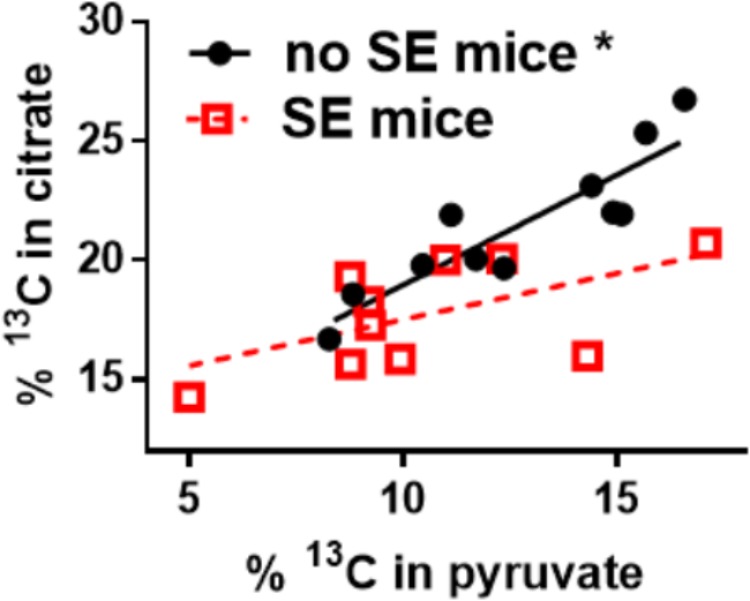
Alteration of ^13^C-pyruvate entry into the TCA cycle in hippocampal tissue of chronically epileptic mice. ^13^C-glucose was injected (i.p.) into mice that were in the chronic epileptic stage of the pilocarpine SE model (SE mice - red open squares) and control no SE mice, which do not show spontaneous seizures (black circles). 15 min later mice were sacrificed and the hippocampi extracted for LCMS-MS analysis. The % enrichment of ^13^C-containing citrate was plotted against the % enrichment of ^13^C-containing pyruvate. A reduction of ^13^C-carbon entry into the Krebs cycle can be observed in the 17% lower hippocampal % ^13^C enrichment in citrate in SE vs. no SE mice (*p* = 0.004, please note that the pyruvate levels were similar). Also, there is a lack of correlation of % ^13^C enrichment in citrate vs. that of pyruvate in SE mice (slope is not significantly different from zero, *p* = 0.08), while in no SE mice there is a correlation (*p* = 0.0002). The lack of correlation indicates that in “epileptic” mice the pyruvate levels do not determine entry into the Krebs cycle, indicating that some other factor is impaired, for example the activity of PDH. Data from (McDonald et al., 2017).

Our recent findings show that the impairment of glucose metabolism observed in epilepsy include mechanisms that result in the reduction of PDH activity. The activities of brain mitochondrial enzymes were quantified in three seizure models. We found that the maximal activities of PDH and some other, but not all mitochondrial enzymes, were consistently reduced. Namely, PDH activities were decreased by 33% in hippocampal extracts from the chronic “epileptic” stage of the pilocarpine mouse model (McDonald et al., 2017), by 43% after a five min flurothyl-elicited seizure (McDonald and Borges, 2017) as well as by 57% during onset of and by 74% 24 h after pilocarpine-induced SE (data unpublished, all *p* < 0.05). Please note, that there is no significant cell loss in the flurothyl model and within the first 24 h after pilocarpine-stimulated SE. The majority of neuronal degeneration is becoming obvious 48 h after SE (Borges et al., 2003). The loss of PDH activity was coupled with a reduction of ^13^C-glucose entry into the TCA cycle after a five min flurothyl-generated seizure (McDonald and Borges, 2017) and in the chronic “epileptic” stage of the pilocarpine mouse model (Smeland et al., 2013; McDonald et al., 2017) and was also previously described in the rat kainate model (Melo et al., 2005). The reduction of ^13^C-carbon entry into the TCA cycle can be observed in the 17% lower hippocampal % ^13^C enrichment of citrate in “epileptic” pilocarpine SE vs. “healthy no SE” mice (*p* = 0.004, **Figure 2**); please note that the total and ^13^C-pyruvate levels were similar between both groups (McDonald et al., 2017). Finally, there was lack of correlation between the % ^13^C enrichment of citrate vs. that of pyruvate in “epileptic” SE mice (slope is not significantly different from zero, *p* = 0.08), while in control “no SE” mice there is a strong correlation (*p* = 0.0002, **Figure 2**) (McDonald et al., 2017). The lack of correlation also indicated that in “epileptic” mice the pyruvate levels do not determine entry into the TCA cycle, indicating and corroborating that the activity of PDH is impaired.

The mechanisms of the decreases in PDH activity and entry of ^13^C-glucose-derived carbons into the TCA cycle were further investigated in the chronic stage of the mouse pilocarpine model and after flurothyl-stimulated seizures. In the pilocarpine model, hippocampal PDH protein levels were reduced by 37% and phosphorylation at Ser300 of the PDH E1α subunit was increased by 52% (Durie et al., 2018). After flurothyl-induced seizures there was a 1.9-fold increase in the phosphorylation of PDH E1α at the serine residue positioned at 232 (McDonald and Borges, 2017). Ser^300^ can be phosphorylated by all four PDH kinase isoforms (PDHK1-4), while Ser232 is specifically phosphorylated by PDHK1 [reviewed by (Rardin et al., 2009)]. Phosphorylation is known to decrease PDH activity and appears to be at least partially responsible for the decreased activity of PDH. Taken together, these data provide evidence that entry of ^13^C-glucose-derived carbon into the TCA cycle and PDH activity are reduced inter- and post-ictally, which can hinder ATP production and normal brain function as well as delay brain recovery after seizures. Decreasing PDH phosphorylation seems to be a promising approach to increase PDH activity and increase seizure thresholds. Unfortunately, chronic oral treatment with the PDHK1-4 inhibitor dichloroacetate was unsuccessful in increasing PDH activity in the chronic pilocarpine mouse model. Also, no anticonvulsant effects were found in a second hit flurothyl test in this chronic model, nor in acute flurothyl or 6 Hz tests (Durie et al., 2018). However, it still needs to be investigated whether this lack of effects are due to insufficient concentrations of dichloroacetate passing the blood brain barrier to reach the affected tissue.

#### Other TCA Cycle and Related Enzymes

Many investigators have studied the effect of SE on TCA cycle enzyme activities. For example, in the first 44 h following SE in rats both aconitase and α-ketoglurate dehydrogenase activity was reduced by approximately 20% in the brain and this reduction was associated with a decrease in reduced glutathione levels (Cock et al., 2002). A similar study showed glutathione levels to decrease in the hippocampus following SE, which was followed by a drop in the activities of aconitase and complex I (Sleven et al., 2006). In our studies, the activities of OGDH in addition to PDH and mitochondrial superoxide dismutase were decreased in mouse hippocampal extracts one day after SE (Tan et al., 2018). Together these studies implicate oxidative stress as one main mediator in limiting enzyme activities.

In the chronic stage of pilocarpine-SE mice, we have not found any signs of oxidative stress, although we reported decreased dehydrogenase activities, including those of PDH, OGDH and GDH. Together with the results from ^13^C-glucose-derived 13-carbon tracing throughout the TCA cycle this shows deficiencies in the TCA cycle energy metabolism, which are likely to contribute to seizure generation (McDonald et al., 2017). Moreover, this loss in TCA cycle activity is expected to impair the generation of lipids and amino acids, which may slow down regeneration, repair and learning, which are all dependent on protein synthesis.

### Electron Transport Chain

Several studies have investigated alterations in the electron transport chain typically together with oxidative stress [please see for review (Rowley and Patel, 2013; Kovac et al., 2017; Pearson-Smith and Patel, 2017)]. For example in humans with TLE, mitochondrial complex I deficiency was found, which is typically regarded as a result of oxidative stress (Kunz et al., 2000). This coincided with increased activities of succinate dehydrogenase staining in CA3 neurons, which may indicate an attempt by the cells to increase energy production by feeding more substrate into complex II to compensate for the complex I deficiency. These studies also support the theory that oxidative stress may contribute to the reduction of activities of TCA cycle enzymes, as many of them are particularly sensitive to oxidation due to their structure.

In contrast, in the chronic phase of the mouse pilocarpine model, our laboratory did not detect any abnormalities in hippocampal mitochondrial respiration states, including respiration linked to ATP synthesis or driven by complex I or II (McDonald et al., 2017). However, one day, but not 2 days, after pilocarpine-induced SE, we have consistently reported reductions in the activities of complex I and II as well as lowered state 2, state 3u mitochondrial respiration and respiration linked to ATP synthesis (Carrasco-Pozo et al., 2015; Tan et al., 2018). This is also found by other laboratories after SE (Chuang et al., 2004) and mentioned in review articles (Rowley and Patel, 2013; Kovac et al., 2017; Pearson-Smith and Patel, 2017). These (transient) impairments are most likely caused by oxidative stress, as we also found transiently increased hippocampal levels of malondialdehyde one day after SE, which were restored 2 days after SE in our model (Carrasco-Pozo et al., 2015).

### Summary of Metabolic Impairments in Epilepsy

The decreased levels in key intermediates of the TCA cycle, as well as the reduction of PDH activity and glucose carbon entry into the TCA cycle are expected to decrease the flux through the TCA cycle, thus limiting the production of the high energy metabolites NADH and FADH_2_. This reduces the substrate availability for the electron transport chain, resulting in decreased ATP production. This corroborates the results from both *in vivo* and *in vitro* studies that have shown a depletion of intracellular ATP (Duffy et al., 1975; Kovac et al., 2012). Similarly, 3-nitropropionic acid, an inhibitor of Complex II which thus impairs ATP synthesis, has been shown to have convulsant activities (Urbanska et al., 1998). In addition, reduced entry of glucose-derived carbons into the TCA cycle may limit the production of lipids and amino acids, thereby slowing down repair processes.

### Metabolic Therapies for the Treatment of Epilepsy

The current anti-seizure drugs typically inhibit neurotransmission, resulting in sedative and cognitive deficits and other side effects, including mood alterations, weight gain and teratogenicity. Please note that by limiting the abnormal electrical activity in the brain and due to sedative side effects, current drugs reduce the need for energy and may thereby further improve the status of an “epileptic” brain. As an alternative to current drug therapies, the ketogenic and derived diets are very effective in pediatric epilepsy (Martin et al., 2016), and have many beneficial cellular effects. It has been well described for over five decades that brain can utilize ketone bodies as an alternate energy substrate to glucose in humans (Owen et al., 1967) and in animals. For example, ketosis proportionately spares glucose utilization in rat brain (Zhang et al., 2013). Here, we discuss several other approaches that are aimed at improving the metabolic deficits in epilepsy summarized above, with an emphasis on even medium chain triglycerides and triheptanoin.

#### TCA Cycle Intermediates

In light of the notion that reduced ATP production and loss of TCA cycle intermediates are associated with seizures, supplementation of TCA cycle substrates have been investigated as a potential therapeutic approach for epilepsy. Pyruvate, the natural substrate for the TCA cycle, can cross the blood brain barrier via monocarboxylate transporters and reduced neuronal cell death in several chronic models of SE in rats (Kim et al., 2007; Carvalho et al., 2011) and abolished epileptic phenotypes in several models (Popova et al., 2017). Injections of a mixture of ascorbic acid, alpha-tocopherol and sodium pyruvate, improved mitochondrial functions and reduced seizures in two chronic mouse models (Simeone et al., 2014). Furthermore a patient with Leigh’s syndrome consuming 500 mg/kg/day of sodium pyruvate experienced a reduction in the occurrence of epileptic seizures (Koga et al., 2012). The anticonvulsant effects are thought to be due to the dual action of pyruvate providing acetyl-CoA and anaplerosis (refilling the TCA intermediate levels) via the carboxylation to oxaloacetate as well as other mechanisms (Popova et al., 2017).

Direct refilling the TCA cycle metabolites via supplementation of either α-ketoglutarate or oxaloacetate attenuated neuronal damage in several rodent seizure models. α-ketoglutarate (500 mg/kg; i.p.) alone decreased the susceptibility to cyanide-provoked seizures (Yamamoto, 1990). Pre-treatment with α-ketoglutarate (2000 mg/k; i.p.) or oxaloacetate (1000 mg/kg; i.p.) was also shown to protect mice from kainic acid-induced seizures and the associated mitochondrial damage and lipid peroxidation (Yamamoto and Mohanan, 2003).

#### Even Medium Chain Triglycerides

The brain can also produce acetyl-CoA directly from C4 ketone bodies and medium chain triglycerides by bypassing the enzymatic reactions of PDH. Originally, ketogenic diets, that increase the plasma levels of ketone bodies, were developed to mimic the beneficial effects of fasting for the treatment of epilepsy. Later, to allow patients to consume higher amounts of carbohydrates and protein, the medium chain triglyceride ketogenic therapy was developed. In this dietary regimen 50–65% of energy intake is from triglycerides of medium chain fats with 6–12 carbons, which are more ketogenic than longer chain fats.

Medium chain triglycerides, usually chemically derived from coconut oil, contain 30–70% trioctanoin and 30–70% tridecanoin, namely the triglycerides of octanoic and decanoic acids (C8 and C10 fatty acids, also called caprylic and carpic acid, respectively). Being tasteless, they can easily be added to regular meals or intragastric/duodenal nutrition. They can be powderised and are used as dietary supplements for patients with metabolic problems or while on ketogenic therapy and athletes in the form of powders, drinks and nutrition bars. Medium chain triglycerides can also be formulated into isotonic emulsions for parenteral nutrition for intensive care patients. After hydrolysis of medium chain triglycerides in the gastro-intestinal tract, the free medium chain fatty acids diffuse directly into blood and mitochondria of all tissues. This is unlike long chain fatty acids, which are much more slowly metabolized, because they are first transported by the lymph and require transport proteins in the blood and for final transport into mitochondria for β-oxidation. Importantly, medium chain fats enter the TCA cycle directly as acetyl-CoA and do not require PDH activity, which is reduced in models after acute and chronic seizures (McDonald and Borges, 2017; McDonald et al., 2017). Also, medium chain fats, especially octanoic acids, are converted by the liver to the C4 ketone bodies β-hydroxybutyrate and acetoacetate, which are taken up into cells by monocarboxylate transporters. We found that feeding 35% of calories (17% w/w diet) of tridecanoin was anticonvulsant in the 6 Hz and flurothyl seizure models, indicating efficacy for focal seizures (Tan et al., 2017). There was a rise in the anti-oxidant activity of plasma in the tridecanoin-fed mice, similar to that observed after treatment with sulforaphane, showing that decanoic acid is indirectly antioxidant. Moreover, we found that oral tridecanoin treatment upregulated the hippocampal mRNA levels of several antioxidant genes, such as Heme oxygenase I (Hmox1) and also Fork-head box O1 (FoxO1) mRNAs. The transcription factor FoxO1 can regulate Hmox1, SOD and catalase expression (Shao et al., 2014). Under oxygen glucose deprivation, neuronal cultures treated with 200 uM decanoic acid reduced the expression of prolyl-hydroxylases 1 and 2 mRNA by 23 and 27% (*p* = 0.028 and 0.047, unpublished). This is expected to stabilize hypoxia-induced factor 1α, which has neuroprotective properties (Puchowicz et al., 2008). These transcriptional changes still need to be verified at the protein level. Yet, they are most likely implicated in tridecanoin’s anti-seizure effects and may protect against damage, because oxidative stress is a well-known contributor to neuronal death (Sciamanna et al., 1992; Di Lisa and Bernardi, 1998).

Medium chain fats appear to be largely β-oxidized by astrocytes (Kuge et al., 1995; Ebert et al., 2003). In an attempt to understand the role of octanoic and decanoic acids as fuels in the brain, we investigated their effects [at 200 μM, a level that has been reported in the plasma of children who had received oral medium chain triglycerides (Sills et al., 1986)] in cultures of neonatal astrocytes. We used a buffer with relatively low glucose and glutamine concentration to mimic their concentrations found in mouse brain extracellular fluid. Relative to 1 mM pyruvate both medium chain fats were able to increase basal respiration and ATP turnover, indicating that they are both excellent substrates for astrocytes. This is also similar to the finding that 6 days decanoic acid treatment increased activity of citrate synthase and complex I in a neuronal cell line (Hughes et al., 2014). Last, we found that decanoic acid increased the mitochondrial proton leak, which under ischaemic conditions limits ROS generation and thus prevents oxidative stress (Tan et al., 2017). The high decanoate levels needed to inhibit AMPA type glutamate receptors (IC50 = 0.5 mM) (Chang et al., 2016) were not reached in mouse brain and appear to be unlikely to be achieved in human brain. In mice fed with 35% tridecanoin we measured 50 μM in plasma and only 1 nmol/g in brain, indicating fast metabolism (Tan et al., 2017). Overall, tridecanoin’s anti-seizure, antioxidant and neuroprotective profile is promising and hence motivates further evaluation.

#### Triheptanoin – An Uneven Medium Chain Triglyceride

**FIGURE 3:**
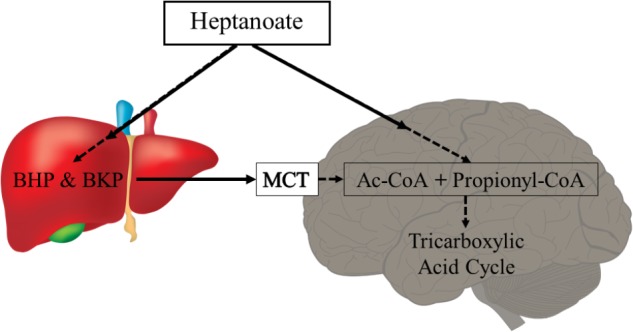
Heptanoate metabolism. Heptanoate can be taken up by the liver to produce the C5 ketone bodies, β-hydroxypentanoate (BHP) and β-ketopentanoate (BKP), which are then released into blood and taken up by cells through monocarboxylate transporters (MCT). It is also likely that heptanoate directly diffuses into cells, including brain cells. In mitochondria, heptanoate metabolites produce acetyl-CoA and anaplerotic propionyl-CoA, which can enter the TCA cycle directly or after carboxylation as succinyl-CoA, respectively.

Metabolism of uneven medium chain fats or C5 ketones does not require PDH activity and can also add substrate into the TCA cycle as succinyl-CoA (anaplerosis). Triheptanoin is an uneven medium chain triglyceride, namely of the C7 heptanoate, which is only found in minute amounts in certain seeds. Therefore, triheptanoin is commercially produced through chemical breakdown of long-chain triglycerides (Semak et al., 2012). Triheptanoin has been used in the treatment of various metabolic disorders associated with metabolic enzyme deficiency and long-chain fat oxidation disorders (Roe et al., 2002, 2008, 2010; Mochel et al., 2005; Roe and Brunengraber, 2015; Gillingham et al., 2017; Vockley et al., 2017). Similar to the even medium chain triglycerides, upon ingestion triheptanoin is hydrolysed into the three heptanoate molecules and glycerol. Heptanoate enters the blood stream and tissues directly via diffusion or is metabolized into the five carbon ketone bodies, β–hydroxypentanoate and β-ketopentanoate via β-oxidation or to glucose via gluconeogenesis in the liver (**Figure 3**) (Kinman et al., 2006). The liver can release the five carbon ketone bodies into the blood, from which they can enter tissues by uptake via monocarboxylate transporters. The C5 ketone bodies and heptanoate are broken into acetyl-CoA and propionyl-CoA molecules in mitochondria. Acetyl-CoA provides direct fuel to the TCA cycle through the condensation with oxaloacetate. In contrast, propionyl-CoA is anaplerotic, as it can be metabolized in the propionyl-CoA carboxylation pathway, whereby it will undergo three conversions by propionyl-CoA carboxylase, methylmalonyl-CoA racemase and methylmalonyl-CoA mutase, to produce succinyl-CoA (Nuutinen et al., 1981; Owen et al., 2002; Martini et al., 2003; Deng et al., 2009; Kasumov et al., 2009). This pathway provided 6% of anaplerosis in the brain of healthy mice (Marin-Valencia et al., 2013). This low percentage still is a significant contribution based on the high rate of TCA cycling (Brunengraber and Roe, 2006). Under conditions of increased need for energy and biosynthetic precursors, including epilepsy, this anaplerotic support may become even more important and potentially may increase.

Providing 35% of calories in the form of triheptanoin oil for 10 days to 3 weeks has been shown to be an anticonvulsant in five different animal models of epilepsy. These models include the corneal kindling model (Willis et al., 2010), second-hit pentylenetetrazole model (Willis et al., 2010), the maximal electroshock threshold test (Thomas et al., 2012), the acute 6 Hz mouse model (McDonald et al., 2014) and a syndrome-specific genetic mouse model of generalized epilepsy with the GABA_A_γ2(R43Q) mutation (Kim et al., 2013).

Oral triheptanoin, unlike trioctanoin, did not alter the pool sizes of glycolytic and TCA cycle intermediates in the hippocampus in healthy mice (McDonald et al., 2014). This suggests that there will be limited side effects in patients treated with triheptanoin. Also, 3 weeks treatment with 35% triheptanoin in mice during the chronic stage of the pilocarpine epilepsy model (the drug was given after SE) did not change the levels of the TCA cycle-derived metabolites, aspartate, glutamate or GABA in the forebrain (Willis et al., 2010). On the other hand triheptanoin prevented loss in the total forebrain pool of malate in the chronic epileptic stage compared to the “healthy” no SE mice (Willis et al., 2010). In addition, in a more recent study triheptanoin attenuated the drop in the percent ^13^C enrichment in the hippocampal formation of “epileptic mice” in malate, citrate and GABA and increased this enrichment in fumarate (Hadera et al., 2014). Taken together this indicates that triheptanoin is anaplerotic in “epileptic” brains. Recently triheptanoin pre-treatment was found to preserve functional mitochondrial parameters, such as respiration linked to ATP synthesis and to prevent the loss of both PDH and OGDH activities in the hippocampal formation 24 h after SE, while SE severity was unaltered. In addition, triheptanoin pre-treatment limited the decreases of the activities of mitochondrial superoxide dismutase and ferric iron reduction of plasma, a marker for plasma anti-oxidant activity, as well as the increase in malondialdehyde levels in mouse hippocampal extracts one day after SE (Tan et al., 2018). Furthermore, *in vitro* OGDH activities in hippocampal extracts from triheptanoin-treated mice were less sensitive to oxidative stress induced by the Fenton reaction. Thus, triheptanoin may be indirectly an antioxidant, which can help to protect the TCA cycle enzyme activities and TCA cycle flux.

A small open label clinical study in 12 children with different types of treatment-resistant epilepsy showed feasibility of adding triheptanoin to a normal diet, with eight children finishing the 3 months treatment phase (Calvert et al., 2018). The main non-serious adverse events were gastro-intestinal problems, such as diarrhea. Seizures were reduced in five children during the treatment phase, although they resumed later in four children during extension of the treatment. Larger studies are needed to evaluate which specific seizure types respond to triheptanoin.

### Summary and Conclusion of Metabolic Treatments

Pre-clinical and clinical studies have defined various metabolic impairments in the chronic epileptic brain. These are specifically targeted by the metabolic treatments discussed, with some treatments showing promising results regarding seizure protection in rodent models, such as pyruvate, tridecanoin and triheptanoin. As these treatments are rapidly metabolized it is difficult to assess kinetics of the active metabolites in plasma and brain. However, the metabolic rate of these alternative fuels may be very different in rodents and humans. In addition, chronic effects on metabolism are likely and may underlie the anti-seizure effects observed. More studies are now needed to assess whether treatments in humans are feasible.

## Author Contributions

TM and KB wrote the manuscript, which was edited by MP. KB designed the figures.

## Conflict of Interest Statement

KB has filed for a patent for the use of triheptanoin in seizure disorders and was granted a patent on the use of triheptanoin in Amyotrophic Lateral Sclerosis. The remaining authors declare that the research was conducted in the absence of any commercial or financial relationships that could be construed as a potential conflict of interest.
